# Targeting the JAK2/STAT3 signaling pathway for chronic pain

**DOI:** 10.14336/AD.2023.0515

**Published:** 2024-02-01

**Authors:** Xin-Yi Dai, Lin Liu, Fan-He Song, Shao-Jie Gao, Jia-Yi Wu, Dan-Yang Li, Long-Qing Zhang, Dai-Qiang Liu, Ya-Qun Zhou, Wei Mei

**Affiliations:** ^1^Department of Anesthesiology, Tongji Hospital, Tongji Medical College, Huazhong University of Science and Technology, Wuhan, China.; ^2^Hubei Key Laboratory of Geriatric Anesthesia and Perioperative Brain Health, Wuhan, China.; ^3^Wuhan Clinical Research Center for Geriatric Anesthesia, Wuhan, China

**Keywords:** JAK2, STAT3, chronic pain, neuroinflammation, microglia, astrocyte

## Abstract

Chronic pain is a notable health concern because of its prevalence, persistence, and associated mental stress. Drugs targeting chronic pain with potent abirritation, and minimal side effects remain unidentified. Substantial evidence indicates that the Janus Kinase 2 (JAK2)/signal transducer and activator of transcription 3 (STAT3) signaling pathway plays a distinct and critical role in different stages of chronic pain. Aberrant activation of the JAK2/STAT3 signaling pathway is evident in multiple chronic pain models. Moreover, an increasing number of studies have demonstrated that the downregulation of JAK2/STAT3 can attenuate chronic pain in different animal models. In this review, we investigated the mechanism and role of the JAK2/STAT3 signaling pathway in modulating chronic pain. The aberrant activation of JAK2/STAT3 can trigger chronic pain by interacting with microglia and astrocytes, releasing proinflammatory cytokines, inhibiting anti-inflammatory cytokines, and regulating synaptic plasticity. We also retrospectively reviewed current reports on JAK2/STAT3 pharmacological inhibitors that demonstrated their significant therapeutic potential in different types of chronic pain. In summary, our results provide strong evidence that the JAK2/STAT3 signaling pathway is a promising therapeutic target for chronic pain.

## 1. Introduction

Chronic pain, such as neuropathic pain from injury or nervous system disease, inflammatory pain, or cancer-induced bone pain, is a severe public health concern [1, 2]. It is a prevalent, persistent, and debilitating condition that worsens patient quality of life [3]. Although morphine is the most commonly administered drug for chronic pain, its side effects seriously limit its clinical utility [4-6]. Thus, it is vital and urgent to elucidate the molecular and cellular mechanisms underlying chronic pain to accelerate the development of new drugs with potent abirritation and minimal side effects as the desired outcome.

The Janus Kinase 2 (JAK2)/signal transducer and activator of transcription 3 (STAT3) pathway plays a pivotal role in the development and maintenance of chronic pain [7-9]. Cytokines can trigger the activation of the JAK2/STAT3 signaling pathway to modulate target gene expression [10]. The JAK2/STAT3 pathway mediates inflammatory and immune responses that eventually participate in neural degeneration, neuroinflammatory responses, synaptic plasticity, and memory formation in the central nervous system (CNS, i.e., the spinal cord and brain) [4, 10-12]. Accumulating basic research using animal models has demonstrated the complex mechanisms and regulation of the JAK2/STAT3 pathway in the pathological and physiological processes of chronic pain. However, the specific molecular and cellular mechanisms involved remain unknown.

In this review, we investigated the mechanism and role of JAK2/STAT3 in modulating chronic pain. We also retrospectively reviewed the current reports on specific pharmacological inhibitors of the JAK2/STAT3 to demonstrate their significant therapeutic potential in alleviating different types of chronic pain.

## 2. Overview of the JAK2/STAT3 signaling pathway

Activated by tyrosine phosphorylation, JAK2 is a non-cross-model tyrosine kinase [13]. The canonical JAK2/ STAT3 pathway is a key mechanism for activating STAT3 transcription [14]. STAT3 is a JAK2 substrate located on chromosome 17q21 and encodes an 89 kDa-length protein [15]. The crucial step of JAK activation involves Janus homology domain 1, which catalyzes the transphosphorylation of Tyr1007 and Tyr1008 (JAK2 tyrosine residues) in the kinase reaction loop. This phosphorylation enables JAK2 to bind to cytokine receptors and numerous signaling molecules that possess the tyrosine kinase Src homology 2 (SH2) domain which attributed to its catalytic domain without SH2 domains [16-18].

To detect extracellular signals, the cytokine binds to its receptors and subsequently triggers receptor molecule dimerization, with receptor-coupled JAKs approaching and transforming into phosphorylated-JAK2 (p-JAK2). To recruit STAT3 protein containing the SH2 domain, activated JAK continuously catalyzes the phosphorylation of Tyr1007 and Tyr1008 on p-JAK2 to form the STAT3 docking site in the cytoplasm. The two monomers reciprocally interact with phospho-tyrosine within the SH2 domains to form Tyr705, a tyrosine residue between SH2 and transcription activation domain phosphorylation [19]. However, owing to the nuclear membrane barrier, a specific transport receptor known as importin transports the STAT3 macromolecule, which is composed of α and β subunits. The homo- or hetero-dimerization of the two STAT3 subunits is guided to combine with importin α through a specific and active motif called the nuclear localization signal on the protein [15]. Subsequently, importin β adheres the protein/importin compound to nuclear pore complexes (NPCs) by interacting with importin α. NPC-related GTPases provide energy to facilitate active transport[20]. Ultimately, activated STAT3 regulates the expression of various target genes by binding to specific promoter sequences [16].

## 3. JAK2/STAT3 signaling pathway is abnormally activated in chronic pain models

Compelling experiments have indicated that the JAK2/STAT3 signaling pathway is aberrantly activated in the sensory nerve soma present in the dorsal root ganglia (DRG), motor neurons in the ventral spinal cord, and red nucleus (RN) in rodents with chronic pain. Recent studies have suggested that the JAK2/STAT3 pathway is abnormally activated in different forms of chronic pain, including neuropathic pain [4,11,14,21-23], inflammatory pain [3, 7, 24, 25], morphine tolerance[4] and bone cancer pain [8, 26, 27].

The JAK2/STAT3 activation occurs following nerve injury and can be triggered by the interleukin-6 (IL-6) family [28]. Dominguez et al. demonstrated that after a spared nerve injury (SNI) phosphorylated-STAT3 (p-STAT3) accumulates in activated microglial cells, which coincides with early and abundant IL-6 mRNA expression in the DRG and rising IL-6 concentrations in the dorsal spinal cord. Notably, the p-STAT3 immunoreactive material (p-STAT3-IR) was assembled chiefly in the superficial and medial laminae (I-V) and was detected in the L5/L6 segments of the spinal cord (sciatic nerve) in rats with spinal nerve ligation (SNL) [29]. Similar results were observed in rats with early stage oxaliplatin-induced acute neuropathic pain, where IL-6 and p-STAT3 levels were markedly increased in the spinal cord [30]. In another study, chronic constriction injury (CCI) surgery significantly increased p-STAT3 levels in the spinal cord. The p-STAT3-IR is also mainly restricted to the L4-L5 spinal dorsal horn (SDH), which corresponds to the central projection area of the respective injured nerves. Evidence indicates that STAT3 may be phosphorylated by signals from central afferent terminals [11, 14]. To comprehensively confirm the mechanisms of the JAK2/STAT3 in the DRG, granulocyte-macrophage colony-stimulating factor (GM-CSF) was used to activate the JAK2 and STAT3 signaling pathways in DRG neurons, ultimately facilitating Nav1.7-1.9 transcription [31].

Mounting evidence supports the anomalous activation of the JAK2/STAT3 in the spinal cord dorsal horn (SCDH). Tian et al. revealed that the p-STAT3 expression in the SCDH and spinal N-methyl-D-aspartate (NMDA)-induced currents were upregulated by leptin treatment in the substantia gelatinosa (lamina II) [32]. In subsequent studies, enhanced p-STAT3-IR was observed in the ipsilateral SDH of rats with SNL [21, 33] as well as in bilateral CCI (bCCI)-induced STAT3 mRNA and p-STAT3 staining in the spinal dorsal cord [11, 34, 35]. To further prove the role of the JAK2/STAT3 in inflammatory pain models, researchers reported that in goat models, 2,4,6-trinitro-benzene-sulfonic acid-ethanol solution treatment increased IL-6, p-JAK2, and p-STAT3 in the nucleus raphe magnus, nucleus tractus solitaries, ventrolateral periaqueductal gray, and dorsal motor nucleus of vagi while increasing their protein and mRNA levels in the SCDH [7]. Similarly, in tumor cell implantation (TCI) models, the upregulated levels of microglial p-JAK2 and p-STAT3 proteins in the spinal cord horn are involved in the development and maintenance of cancer-induced bone pain (CIBP) [8].

Substantial evidence has shown that the red nucleus (RN) JAK2/STAT3 is aberrantly activated in the modulation of chronic pain by regulating cytokines. RN IL-6 induces the production of tumor necrosis factor-α (TNF-α) and interleukin 1β (IL-1β) that mediate the maintenance of neuropathic pain by activating the JAK2/STAT3 [36]. Ding et al. discovered an increase in p-STAT3 protein and IL-6 levels in the RN on the side contralateral to the nerve injury site after SNI [37]. Additionally, p-STAT3 upregulation mediated by IL-1β in RN neurons and astrocytes is involved in pain modulation and tactile allodynia [38]. Furthermore, the RN interleukin 33 (IL-33) exerts an analgesic effect by predominantly upregulating the protein expression of RN p-JAK2 and p-STAT3 in the early stages of SNI-induced mono-neuropathic pain [39]. These results provide central evidence suggesting a potential association between a dysregulated JAK2/STAT3 signaling pathway and the RN in chronic pain.

## 4. Upstream regulatory factors of the JAK2/STAT3 signaling pathway in chronic pain

### 4.1 Microglia activation mediates the JAK2/STAT3 signaling pathway to regulate chronic pain

Robust evidence has unequivocally confirmed that microglia activation in the CNS participates in the early stage and progression of chronic pain [3, 28, 29, 40-42]. As the initial line of immune defense, microglial cells, known as “pathological effectors and amplifiers,” in the CNS use highly motile processes to routinely monitor and investigate their surrounding territory [28]. Microglial cells must be appropriately activated to promote optimal body function. By the way, microglia undergo apoptosis during nerve injury [40]. After damage to the peripheral nervous system, microglia are activated and polarized toward the M1 phenotype to release inflammatory cytokines, such as IL-1β, IL-6, and TNF-α, in the early stages. The associated functional and morphological changes, including inflammatory gene upregulation, transcriptional activation, microgliosis, and migration, ultimately lead to the onset of neuropathic pain [28, 43, 44]. Li et al. demonstrated that owing to IL-6 production induced by nerve injury in the DRG, the central terminals of primary afferents may transport cytokines for release, which stimulates the microglial JAK2/STAT3 signaling pathway and induces neuropathic pain. Furthermore, these cytokines induce microglial cells and neurons to release more activating substances, such as prostaglandins, reactive oxygen species, proinflammatory factors, nitric oxide, and adenosine triphosphate, which enhance neuropathic pain [45]. Collectively, these results suggest that spinal microglia activation is a vital and pivotal mediator that significantly affects the development and maintenance of chronic pain.

Current research has revealed that the microglial JAK2/STAT3 signaling pathway plays a crucial role in the process of chronic pain. Intrathecal treatment with lncRNAs downregulated in liver cancer stem cell (DILC) siRNA in bCCI rats alleviates mechanical allodynia and thermal hyperalgesia by promoting the suppressor of cytokine signaling 3 (SOCS3) expression and downregulating the JAK2/STAT3 pathway leading to enhanced microglial viability, reduced cell apoptosis, and inflammation [40]. Chen et al. first revealed that TCI-induced mechanical allodynia was mitigated by upregulation of the spinal microglial JAK2/STAT3 pathway by chronic intraperitoneal administration of sinomenine (a potent inhibitor of microglial activation) in a CIBP female rat model [8]. Furthermore, the P2Y13 receptor is a neurotransmitter receptor of spinal microglial cells, and intrathecal administration of MRS2211, a P2Y13 receptor antagonist, attenuated IL-6/JAK2/ STAT3-induced mechanical allodynia in the dorsal spinal cord in an early-stage painful diabetic neuropathy (DNP) rat model [23]. This result is similar to those reported by Guo et al. and Sun et al. [28, 41]. Additionally, Yu et al. elucidated that intraperitoneal muscone treatment effectively suppressed mechanical allodynia and thermal hyperalgesia induced by complete Freund’s adjuvant (CFA). This was attributed to the inhibitory effect of the nicotinamide adenine dinucleotide phosphate oxidase 4 (NOX4)/JAK2-STAT3 pathway-mediated inflammatory response associated with microglia activation in male mice[3]. A similar result was reported, in which the oral administration of 3,5-dicaffeoylquinic acid, a pro-inflammatory cytokine inhibitor, attenuated CFA-induced hyperalgesia by inhibiting microglial activation and upregulating autophagy in the spinal cord through the inhibition of monocyte chemotactic protein 3 (MCP3)/JAK2/STAT3 signaling [25].

As previously stated, convincing research has demonstrated that the microglial JAK2/STAT3 signaling pathway maintains the regulation of chronic pain via transcription and expression of genes, such as IL-6, SOCS3, NOX4, and MCP3. Furthermore, microglial JAK2/STAT3 pathway activity may regulate chronic pain by directly affecting the vitality of astrocytes and neurons and modulating spinal cord plasticity and remodeling [8].

### 4.2 Astrocyte activation mediates the JAK2/STAT3 signaling pathway to regulate chronic pain

Similar to microglia, astrocyte activation in the spinal cord plays a pivotal role in initiating and maintaining chronic pathological pain by upregulating glial fibrillary acidic protein (GFAP, an astrocytic marker) and cell hypertrophy [27, 46, 47]. Existing literature suggests that astrocyte activation is dependent on the phosphorylation state of the JAK2/STAT3 signaling pathway [21, 27, 48-50]. Tang et al. validated that intrathecal treatment with triptolide, which has vigoroso anti-inflammatory and immunosuppressive properties, ameliorated SNL-induced mechanical allodynia associated by inhibiting the JAK2/STAT3 signaling pathway, consequently reducing spinal astrocyte activation in male rats [21]. Similar results showed that SNI-induced mechanical and cold allodynia was notably relieved via intrathecal injection of ST2-neutralizing antibody or ST2 gene knockout, which inhibited astroglial JAK2/STAT3 cascade activation in adult male mice [33]. In a subsequent study, Liu et al. reported a dose-dependent attenuation of SNI-induced mechanical and cold allodynia in a mouse model through intraperitoneal treatment with curcumin, which displays strong anti-inflammatory activity, by inhibiting the JAK2/STAT3 cascade activation in spinal astrocytes [22]. Growing evidence has shown that astroglial JAK2/STAT3 activation is vital for pain regulation in SNI models. Nevertheless, several studies have demonstrated the critical role of astrocytes in other chronic pain models. For instance, miR-135-5p has a neuroprotective function attributed to the suppression of cellular apoptosis. Liu et al. showed that intrathecal administration of miR-135-5p potentially ameliorated bone cancer pain (BCP) in mouse models by blocking astrocyte activation and the JAK2/STAT3 signaling pathway [27]. Collectively, these crucial findings highlight the significant role of astroglial JAK2/STAT3 activation in chronic pain and underscore the need for further studies to elucidate the underlying potential molecular and cellular mechanisms.

### 4.3 α7nAChR activation inhibits the JAK2/STAT3 signaling pathway to modulate chronic pain

The “cholinergic anti-inflammatory pathway” (CAP) is proposed to modulate the immune and nervous systems through acetylcholine (ACh) binding to the alpha-7 nicotinic acetylcholine receptor (α7nAChR). α7nAChR is widely distributed in the spinal cord and DRG of spinal pain transmission pathways, where it modulates chronic pain by inhibiting phosphorylation of its downstream molecules JAK2/STAT3 and release of the proinflammatory cytokines IL-1β, IL-6, and TNF-α [51-53]. In an adult male SNI rat model, 2 Hz electroacupuncture (EA) treatment was found to significantly activate α7nAChR and inhibit the JAK2/STAT3 signaling, which then rebalanced the relationship between proinflammatory and anti-inflammatory cytokines in the DRG [51]. Furthermore, Wang et al. reported that 2 Hz EA ameliorated mechanical hypersensitivity and downregulated p-JAK2, p-STAT3, and IL-6 expression in SNI rats, which could be suppressed by the intrathecal administration of the α7nAChR antagonist alpha-bungarotoxin (α-Bgtx) [52]. Xie et al. also provided similar evidence [53]. These studies indicate that the JAK2/STAT3 pathway mediates the CAP via α7nAChR-mediated neuroinflammatory responses to regulate SNI-induced chronic pain. As expected from the aforementioned reports, the CAP presents a promising avenue for managing neuropathic pain (NP).

The expression, distribution and cellular localization of the upstream regulatory factors of Janus Kinase 2 (JAK2)/signal transducer and activator of transcription 3 (STAT3) signaling pathways in chronic pain are summarized in Table 1.

## 5. Downstream regulatory factors of the JAK2/STAT3 signaling pathway in chronic pain

### 5.1 JAK2/STAT3 signaling pathway releases proinflammatory cytokines IL-6, IL-1β, TNF-α, IL-33, and SOCS3 to modulate chronic pain

Numerous inflammatory mediators participate in the neuroimmune response, which may be indispensable in chronic pain regulation. Among them, proinflammatory cytokines, including IL-6, IL-1β, and TNF-α, play a key role in the neuroimmune response induced by SNI and are implicated in the genesis of neuropathic pain [51, 54-56]. Numerous models have been established to investigate the mechanisms by which inflammatory cytokines affect chronic pain through the JAK2/STAT3 pathway.

#### 5.1.1 IL-6

The cytokine IL-6 is considered to be proinflammatory and has been reported to play a significant role in the maintenance of NP [21]. IL-6 primarily elicits its biological effects on target cells by interacting with the non-signaling membrane-bound IL-6 receptor (mIL-6R). The resulting IL-6 and mIL-6R complex promotes dimerization and subsequently activates intracellular signaling, including the JAK2/STAT3, by binding to the signal-transducing membrane protein gp130 [57]. Dominguez et al. showed that the intrathecal administration of an anti-rat IL-6 antibody to SNI male rats decreased p-STAT3 levels. Furthermore, a large amount of SNI-induced endogenous IL-6 regulates the generation of ipsilateral mechanical and thermal hypersensitivity and contralateral mechanical allodynia by the JAK2/STAT3 signal transduction pathway in male rats [29]. These findings suggest that IL-6 may exacerbate chronic pain by activating the JAK2/STAT3 pathway. In support of this idea, numerous animal models for chronic pain have been established to investigate the possible mechanisms of IL-6/JAK2/STAT3 signalling, including neuropathic pain [37, 52], inflammatory pain [7], and diabetic neuropathic pain [23]. Moreover, oral treatment with PCC0208009, an indirect indoleamine 2, 3-dioxygenase 1(IDO1) inhibitor, markedly alleviated mechanical allodynia and thermal hyperalgesia in rats with SNL without producing unwanted side effects by inhibiting the IL-6-JAK2/STAT3-IDO1-general control nonderepressible 2 (GCN2)-IL-6 pathway in the anterior cingulate cortex (ACC) and amygdala [58]. As mentioned above, these studies have shown that IL-6 plays a dual role in the regulation of the JAK2/STAT3 in chronic pain models, which do not only activate the JAK2/STAT3, but also be released induced by the JAK2/STAT3.

**Table 1:** Expression, distribution and cellular localization of the upstream regulatory factors of Janus Kinase 2 (JAK2)/signal transducer and activator of transcription 3 (STAT3) signaling pathways in chronic pain.

JAK2/STAT3 signaling pathways	Model	Expression	Distribution	Cellular localization	References
**p-JAK2**	SNL-induced neuropathic pain rats	SDH↑	/	Astrocytes	[24]
		ACC and amygdala↑	/	Astrocytes	[48]
		Spinal cord↑	/	/	[31]
	SNI-induced neuropathic pain rats	DRG↑	/	/	[45]
		Spinal cord↑	/	/	[47]
		RN↑	RN	/	[41]
	SNI-induced neuropathic pain mice	Spinal cord↑	Dorsal horn	Astrocytes	[35]
		Spinal cord↑	/	Astrocytes	[25]
		Spinal cord↑	/	Microglial cells	[43]
	bCCI-induced neuropathic pain rats	Spinal cord↑	Dorsal horn	/	[11]
		Spinal cord↑	/	/	[42]
	CCI-induced neuropathic pain rats	Spinal cord↑	Spinal dorsal horn	/	[36]
	Morphine-induced analgesic tolerance mice	DRG↑	/	/	[4]
	TNBS-treated visceral hypersensitivity goats	PAG-RVM-SCDH axis↑	vlPAG, NRM, SCDH, NTS, DMV	/	[7]
	TCI-induced bone cancer pain rats	Spinal cord↑	Spinal cord horn	Microglial cells	[8]
		SDH↑	/	Neurons	[29]
		DRG↑	DRG: small-sized neurons	DRG neurons	[33]
	TCI-induced bone cancer pain mice	Spinal cord↑	/	Astrocytes	[30]
	STZ-induced diabetic neuropathic pain rats	Spinal cord↑	Dorsal spinal cord	/	[26]
	CFA-induced inflammatory pain mice	Spinal cord↑	/	/	[3]
**p-STAT3**	SNL-induced neuropathic pain rats	Spinal cord↑	Dorsal spinal cord: the superficial and medial laminae (I-V) ventral horn	Microglial cells	[32]
		ACC and amygdala↑	/	Astrocytes	[48]
		Spinal cord↑	/	/	[31]
		SDH↑	/	Astrocytes	[24]
	SNI-induced neuropathic pain rats	RN↑	/	/	[39, 41]
		DRG↑	/	/	[45]
		Spinal cord↑	/	/	[47]
	SNI-induced neuropathic pain mice	Spinal cord↑	/	Microglial cells	[43]
		Spinal cord↑	Spinal dorsal horn	Astrocytes	[35]
		Spinal cord↑	/	Astrocytes	[25]
	CCI-induced neuropathic pain rats	Spinal cord↑	Spinal cord: the superficial and medial laminae (I-V)	Astrocytes	[14]
		Spinal cord↑	Spinal dorsal horn	/	[36]
		Spinal cord↑	/	/	[37]
	bCCI-induced neuropathic pain rats	Spinal cord↑	Dorsal horn	/	[11]
		Spinal cord↑	/	/	[42]
	TNBS-treated visceral hypersensitivity goats	PAG-RVM-SCDH axis↑	vlPAG, NRM, SCDH, NTS	/	[7]
	TCI-induced bone cancer pain rats	Spinal cord↑	Spinal cord horn	Microglial cells	[8]
		SDH↑	/	Neurons	[29]
		DRG↑	DRG: small-sized neurons	DRG neurons	[33]
	TCI-induced bone cancer pain mice	Spinal cord↑	/	Astrocytes	[30]
	Leptin-induced neuropathic pain rats	Spinal cord↑	Dorsal horn: lamina II	neurons	[34]
	IL-1β-mediated pain rats	RN↑	/	RN: neurons and astrocytes	[40]
	Oxaliplatin-induced acute neuropathic pain rats	Spinal cord↑	/	/	[13]
	STZ-induced diabetic neuropathic pain rats	Spinal cord↑	Dorsal spinal cord	/	[26]
	HFSD-induced DNP rats	SDH↑	/	Microglial cells	[44]
	CFA-induced inflammatory pain mice	Spinal cord↑	/	/	[3]
**α7nAchR**	SNI-induced neuropathic pain rats	DRG↓	/	/	[45]
	tMCAO-induced I/RI rats	Cerebral cortex↓	Cerebral cortex: in the ischemic penumbra area	/	[50]

Abbreviations: α7nAchR: alpha-7 nicotinic acetylcholine receptor; ACC: anterior cingulate cortex; bCCI: bilateral chronic constriction injury; CCI: chronic constriction injury of the sciatic nerves; CFA: complete Freund's adjuvant; DMV: dorsal motor nucleus of vagi; DNP: painful diabetic neuropathy; DRG: dorsal root ganglia; encephalomyelitis; HFSD: high-fat-sugar diet; I/RI: ischemia/reperfusion injury; NRM: nucleus raphe magnus; NTS: nucleus tractus solitaries; PAG: periaqueductal gray; RMC: RN magnocellular; RN: red nucleus; RVM: rostral ventromedial medulla; SCDH: spinal cord dorsal horn; SDH: spinal dorsal horn; SNI: spared nerve injury; SNL: spinal nerve ligation; STZ: streptozotocin; TCI: tumor cell implantation; tMCAO: transient middle cerebral artery occlusion; TNBS: 2,4,6-trinitro-benzene-sulfonic acid

#### 5.1.2 IL-1β

Notably, other proinflammatory cytokines also play diverse roles in different stages of chronic pain. Tang et al. indicated that IL-1β and TNF-α participate in the initiation of neuropathic pain [21]. Liu et al. discovered that intrathecal injection of AG490 significantly restrained the synthesis of pro-IL-1β and maturation of IL-1β, thus attenuating SNI-induced mechanical and cold allodynia in male mice [22]. In another study, intracerebral injection of AG490 into the RN before administering recombinant rat IL-1β completely suppressed IL-1β-evoked contralateral tactile allodynia in RN neurons and astrocytes [38]. DNP and BCP in adult rats are attenuated by inhibiting the JAK2/STAT3 cascade activation and downregulating IL-1β expression [23, 26]. Taken together, the results from different chronic pain models provide solid evidence that IL-1β is involved in the development of chronic pain.

#### 5.1.3 TNF-α and crosstalk between various inflammatory cytokines in pain

To further elucidate the role of TNF-α and the crosstalk between various inflammatory cytokines via the JAK2/STAT3 in chronic pain, an increasing number of animal models have been established to investigate these pathological and physiological processes. Tang et al. uncovered that intrathecal treatment with triptolide attenuated mechanical allodynia in SNL male rats, coinciding with the suppression of the JAK2/STAT3 pathway and the downregulation of IL-6, IL-1β, and TNF-α in the ipsilateral SDH[21]. Similar results were demonstrated by Liu et al. and Wang et al.[33, 35, 40]. These substantial findings demonstrate that TNF-α can regulate chronic pain induced by the JAK2/STAT3 pathway in accordance with other proinflammatory cytokines.

#### 5.1.4 IL-33

The biological effects of IL-33 are facilitated by its interaction with its receptor complex ST2. Liu et al. validated the efficacy of intrathecal treatment of adult male mice with either an ST2-neutralizing antibody or an ST2 gene knockout, which alleviated the development and maintenance of SNI-induced mechanical and cold allodynia by restraining astroglial JAK2-STAT3 cascade activation [33]. In a separate study, the intrarubral injection of AG490 into the RN after SNI and before IL-33 treatment relieved SNI-induced mono-neuropathic pain and IL-33-evoked mechanical hypersensitivity in male rats [39]. Collectively, these results reveal that IL-33 within the RN plays a crucial role in the development and maintenance of chronic pain.

#### 5.1.5 SOCS3

The suppressor of cytokine signaling 3 (SOCS3) is a target gene of the STAT3 transcription factor, which has been identified as an essential player in chronic pain through its feedback regulation of the JAK2/STAT3 pathway. Thus, SOCS3 mRNA serves as an index of STAT3 activity [11, 14, 21, 29]. Wang et al. showed that intrathecal injection of AG490 or S3I-201 into adult male rats with CCI significantly reduced mechanical allodynia and spinal neuroinflammation. The study also revealed an increase in the mRNA concentrations of SOCS1 and SOCS3, but not of SOCS2 [14]. Additionally, there was a remarkable reduction in SOCS3 mRNA levels and remission of ipsilateral mechanical allodynia and thermal hyperalgesia caused by AG490 intrathecal treatment in rats with SNL [29]. In summary, these reports validated that SOCS3 relieves neuroinflammation and related chronic pain by regulating the JAK2/STAT3 signaling pathway. Similar results were observed in studies conducted by Liu et al., Tang et al., and Xue et al. [11, 21, 33], which demonstrated that the variation in SOCS3 mRNA and protein levels was consistent with that of the JAK2/STAT3. Compared with the above results, Liu et al. revealed that intrathecal administration of DILC siRNA markedly led to a promotion in mechanical allodynia and thermal and cold hyperalgesia, which was attributed to the suppression of the SOCS3/JAK2/STAT3 pathway in bCCI male rats and a subsequent reduction in the production of IL-1β, IL-6, and TNF-α in the microglia [40]. This outcome may be explained by the negative feedback effect of SOCS3 on the JAK2/STAT3 pathway. As previously mentioned, SOCS3 plays a pivotal and dual role in the feedback effect of the JAK2/STAT3 pathway by regulating the pathological and physiological processes of chronic pain.

### 5.2 JAK2/STAT3 signaling pathway inhibits anti-inflammatory cytokines IL-10 and TGF-β to modulate chronic pain

Recent studies have also shed light on the role of anti-inflammatory cytokines in the development of chronic pain, which may interact with proinflammatory cytokines through the JAK2/STAT3 pathway. According to Wang et al., interleukin 10 (IL-10) is a potent anti-inflammatory cytokine that promotes its anti-inflammatory effects in NP. It has been reported that 2 Hz EA administration notably upregulates IL-10 levels and reduces p-JAK2, pSTAT3, IL-1β, and IL-6 expression in the DRG of rats with SNI [51]. Otherwise, intrarubral treatment with AG490 promoted the expression of transforming growth factor-β (TGF-β) and IL-10 while inhibiting the upregulation of TNF-α, IL-6, and IL-1β in the contralateral SDH(L4-L6) in male rats with IL-6-evoked tactile allodynia [59]. This evidence provides insights into the mechanism by which anti-inflammatory cytokines ameliorate chronic pain via the JAK2/STAT3 and crosstalk with proinflammatory cytokines.

Taken together, the manifestation of pro-inflammatory cytokines in conjunction with the lack of anti-inflammatory cytokines facilitates an imbalance in the cytokine microenvironment, which mediates chronic pain sensitization by modulating the JAK2/STAT3 expression and translation levels.

### 5.3 JAK2/STAT3 signaling pathway releases chemokines to modulate chronic pain

C-C motif ligand 2 (CCL2), a member of the CC chemokine family, triggers the activation of spinal microglia. Several studies have shown that CCL2 neutralizing antibodies or CCL2 receptor (CCR2) antagonists effectively prevent neuropathic pain similar to microglial inhibitors. Liu et al. reported that DILC siRNA markedly alleviated mechanical allodynia and thermal and cold hyperalgesia in bCCI rats and downregulated the expression of integrin alpha M (ITGAM), cyclooxygenase-2 (COX2) and CCL2, which are downstream genes of STAT3 [40]. Additionally, administration of an ST2-neutralizing antibody intrathecally or ST2 gene knockout remarkably ameliorated SNI-induced mechanical and cold allodynia and significantly inhibited the expression of the spinal proinflammatory chemokine CCL2 by inhibiting the astroglial JAK2-STAT3 cascade [33]. These results imply that CCL2 may play a pivotal role in the development of chronic pain via the microglial and astroglial JAK2/STAT3 signaling pathways.

**Table 2:** Expression, distribution and cellular localization of the downstream regulatory factors of Janus Kinase 2 (JAK2)/signal transducer and activator of transcription 3 (STAT3) signaling pathways in chronic pain.

JAK2/STAT3 signaling pathways	Model	Expression	Distribution	Cellular localization	References
**IL-6**	SNL-induced neuropathic pain rats	Spinal cord and DRG↑	Dorsal spinal cord and DRG	/	[32]
		SDH↑	/	Astrocytes	[24]
		Spinal cord↑	/	/	[31]
		ACC and amygdala↑	/	Astrocytes	[48]
	SNI-induced neuropathic pain rats	DRG↑	/	/	[45]
		RN↑	RN: especially RMC	/	[39]
		Spinal cord↑	/	/	[47]
	SNI-induced neuropathic pain mice	Spinal cord↑	Spinal dorsal horn	Astrocytes	[35]
		Spinal cord↑	/	Microglial cells	[43]
	CCI-induced neuropathic pain rats	Spinal cord↑	Spinal cord: the superficial and medial laminae (I-V)	Astrocytes	[14]
	TNBS-treated visceral hypersensitivity goats	PAG-RVM-SCDH axis↑	vlPAG, NRM, SCDH, NTS, and DMV	/	[7]
	oxaliplatin-induced acute neuropathic pain rats	Spinal cord↑	/	/	[13]
	STZ-induced diabetic neuropathic pain rats	Spinal cord↑	Dorsal spinal cord	/	[26]
	tMCAO-induced I/RI rats	Cerebral cortex↑	Cerebral cortex: in the ischemic penumbra area	/	[50]
	Interleukin-6-evoked tactile allodynia rats	SDH↑	/	/	[49]
	HFSD-induced DNP rats	SDH↑	/	Microglial cells	[44]
**IL-1β**	SNL-induced neuropathic pain rats	SDH↑	/	Astrocytes	[24]
		Spinal cord↑	/	/	[31]
		ACC and amygdala↑	/	Astrocytes	[48]
	SNI-induced neuropathic pain rats	DRG↑	/	/	[45]
		RN↑	RN: especially RMC	/	[38]
	SNI-induced neuropathic pain mice	Spinal cord↑	Spinal dorsal horn	Astrocytes	[35]
		Spinal cord↑	/	Astrocytes	[25]
	CCI-induced neuropathic pain rats	Spinal cord↑	Spinal cord: the superficial and medial laminae (I-V)	Astrocytes	[14]
		Spinal cord↑	/	/	[37]
	TCI-induced bone cancer pain rats	SDH↑	/	Neurons	[29]
	TCI-induced bone cancer pain mice	Spinal cord↑	/	Astrocytes	[30]
	rrIL-1β-induced neuropathic pain rats	RN↑	/	RN neurons and astrocytes	[40]
	STZ-induced diabetic neuropathic pain rats	Spinal cord↑	Dorsal spinal cord	/	[26]
	tMCAO-induced I/RI rats	Cerebral cortex↑	Cerebral cortex: in the ischemic penumbra area	/	[50]
	Interleukin-6-evoked tactile allodynia rats	SDH↑	/	/	[49]
**TNF-α**	SNL-induced neuropathic pain rats	SDH↑	/	Astrocytes	[24]
		Spinal cord↑	/	/	[31]
	SNI-induced neuropathic pain rats	RN↑	RN: especially RMC	/	[38]
		RN↑	/	RN neurons	[41]
	SNI-induced neuropathic pain mice	Spinal cord↑	Spinal dorsal horn	Astrocytes	[35]
	CCI-induced neuropathic pain rats	Spinal cord↑	Spinal cord: the superficial and medial laminae (I-V)	Astrocytes	[14]
		Spinal cord↑	/	/	[37]
	bCCI-induced neuropathic pain rats	Spinal cord↑	/	Microglial cells	[42]
	TCI-induced bone cancer pain mice	Spinal cord↑	/	Astrocytes	[30]
	tMCAO-induced I/RI rats	Cerebral cortex↑	Cerebral cortex: in the ischemic penumbra area	/	[50]
	Interleukin-6-evoked tactile allodynia rats	SDH↑	/	/	[49]
**IL-33**	SNI-induced neuropathic pain mice	Spinal cord↑	Spinal dorsal horn	Astrocytes	[35]
		RN↑	/	neurons, oligodendrocytes, and microglia	[41]
**GM-CSF**	TCI-induced bone cancer pain rats	DRG↑	DRG: small-sized neurons	DRG neurons	[33]
**IL-10**	SNI-induced neuropathic pain rats	DRG↓	/	/	[45]
	SNL-induced neuropathic pain rats	Spinal cord↓	/	/	[31]
	Interleukin-6-evoked tactile allodynia rats	SDH↓	/	/	[49]
**TGF-β**	Interleukin-6-evoked tactile allodynia rats	SDH↓	/	/	[49]
	SNL-induced neuropathic pain rats	Spinal cord↓	/	/	[31]
**CCL2**	SNI-induced neuropathic pain mice	Spinal cord↑	/	/	[35]
**SOCS3**	SNL-induced neuropathic pain rats	Spinal cord↑	Dorsal spinal cord	/	[32]
		SDH↑	/	Astrocytes	[24]
	SNI-induced neuropathic pain mice	Spinal cord↑	Spinal dorsal horn	Astrocytes	[35]
	CCI-induced neuropathic pain rats	Spinal cord↑	Spinal cord: the superficial and medial laminae (I-V)	Astrocytes	[14]
	bCCI-induced neuropathic pain rats	Spinal cord↑	Dorsal spinal cord	/	[11]
		Spinal cord↑	/	/	[42]
**p-NR2B**	HFSD-induced DNP rats	SDH↑	/	Microglial cells	[44]
**NMDAR**	Leptin-induced neuropathic pain rats	Spinal cord↑	Dorsal horn: lamina II	neurons	[34]

Abbreviations: ACC: anterior cingulate cortex; bCCI: bilateral chronic constriction injury; CCI: chronic constriction injury of the sciatic nerves; CCL2: (C-C motif) ligand 2; CFA: complete Freund's adjuvant; DMV: dorsal motor nucleus of vagi; DNP: painful diabetic neuropathy; DRG: dorsal root ganglia; encephalomyelitis; GM-CSF: granulocyte-macrophage colony stimulating factor; HFSD: high-fat-sugar diet; I/RI: ischemia/reperfusion injury; IL-1β: interleukin 1β; IL-6: interleukin 6; IL-10: interleukin 10; IL-33: interleukin 33; NMDAR: N-methyl-D-aspartate receptor; NR2B: NMDA receptor 2B; NRM: nucleus raphe magnus; NTS: nucleus tractus solitaries; PAG: periaqueductal gray; RMC: RN magnocellular; RN: red nucleus; RVM: rostral ventromedial medulla; SCDH: spinal cord dorsal horn; SDH: spinal dorsal horn; SNI: spared nerve injury; SNL: spinal nerve ligation; SOCS3: suppressor of cytokine signaling 3; STZ: streptozotocin; TCI: tumor cell implantation; TGF-β: transforming growth factor-β; tMCAO: transient middle cerebral artery occlusion; TNBS: 2,4,6-trinitro-benzene-sulfonic acid; TNF-α: tumor necrosis factor-α.

### 5.4 JAK2/STAT3 signaling pathway activates NMDA receptors to modulate chronic pain

Several reports have provided solid evidence that NMDA receptor 2 B (NR2B)-containing spinal NMDA receptors (NMDARs) in the superficial SDH, such as the NR2B subunit, are critical contributors to peripheral and central sensitization, pain transmission, and development of chronic pain [21, 32]. The JAK2/STAT3 cascade plays a critical role in NMDAR-dependent pain transmission and NP behaviors [23, 33, 45].

In one study, intrathecal MK-801 (a noncompetitive NMDAR antagonist) not only inhibited thermal hyperalgesia, mechanical allodynia, and NMDA-induced pain behaviors but also downregulated the expression of NMDARs and pSTAT3 in the SDH of leptin-induced adult male rats. This study further reported that AG490 decreases the leptin-induced enhancement of NMDA currents in rat dorsal horn neurons [32]. In another study, SNL-induced mechanical allodynia and the JAK2/STAT3 signaling pathway activation were inhibited in SDH astrocytes by suppressing the upregulation of proinflammatory cytokines following intrathecal administration of triptolide (T10), which subsequently suppressed the expression of p-NMDAR in male rats [21]. These results are in accordance with the perspective that NMDAR regulate peripheral sensitization and chronic pain, probably by modulating NMDA currents and inflammatory cytokines induced by the astroglial JAK2/STAT3 pathway.

Furthermore, NMDAR activation is a vital pathogenesis of DNP and can further induce CNS sensitization[45]. Zhou et al. reported that intrathecal MRS2211 (P2Y13 receptor antagonist) not only attenuated mechanical allodynia and downregulated IL-1β and IL-6 levels, which whereafter inhibited the activation of the JAK2/STAT3 signaling pathway but also decreased central sensitization by inhibiting NR2B-containing NMDAR phosphorylation in dorsal spinal cord neurons in rats with early-stage DNP [23]. Additionally, caveolin-1 (CAV-1) is an essential gene targeting STAT3, which affects neuronal plasticity and receptor transport to modulate NR2B-NMDAR. Li et al. showed that the intrathecal injection of AG490 downregulated the abnormal expression of t-CAV-1, p-NR2B, p-JAK2, and p-STAT3 in activated microglia and mitigated pain in the SDH of rats with DNP [45]. Collectively, these results provide compelling evidence that microglial JAK2/STAT3 signaling triggers NP by activating the CAV-1-NR2B pathway.

### 5.5 JAK2/STAT3 signaling pathway mediates upregulation of Nav1.7-1.9 channels to modulate GM-CSF-induced pain

GM-CSF is one of the most general growth factors in the blood. In vitro studies have reported that GM-CSFR activation can stimulate cell signaling pathways, including the JAK2/STAT3 pathway, by modulating gene expression. JAK2/STAT3 has been reported to facilitate the transcription of Nav1.7-1.9 in DRG neurons. Zhang et al. found that targeted knockdown of either Nav1.7-1.9 or the JAK2/STAT3 in DRG neurons attenuated GM-CSF-induced mechanical and thermal hypersensitivity in male rats with BCP [31]. Taken together, these results prove that the JAK2/STAT3-mediated upregulation of Nav1.7-1.9 channels is a pivotal signaling pathway in the development of GM-CSF-induced pain.

The expression, distribution and cellular localization of the downstream regulatory factors of Janus Kinase 2 (JAK2)/signal transducer and activator of transcription 3 (STAT3) signaling pathways in chronic pain are summarized in Table 2.

## 6. Therapeutic potential and clinical application of pharmacological JAK2/STAT3 inhibitors in chronic pain

Multiple pharmacological inhibitors of the JAK2/STAT3 have been reported to have therapeutic potential against different types of chronic pain, such as AG490, WP1066, and S3I-201. However, the current research hotspot is AG490. AG490 is a specific and potent inhibitor of the JAK2/STAT3 signaling that decreases neurological dysfunction and inhibits JAK2 and STAT3 phosphorylation during central inflammation [14, 30, 60, 61]. Studies have shown that different injection routes may modulate chronic pain through different mechanisms. In an adult male rat model of peripheral nerve injury, Dominguez et al. demonstrated that intrathecal administration of AG490 attenuated early-stage ipsilateral mechanical allodynia and thermal hyperalgesia by suppressing the microglial SOCS3/JAK2/ STAT3 signaling pathway [29]. Furthermore, intraperitoneal AG490 administration ameliorates cold and mechanical allodynia in rats with oxaliplatin-induced acute neuropathic pain by inhibiting the activation of the JAK2/STAT3 signaling and suppressing IL-6 expression [30]. Furthermore, intrathecal or intrarubral injection of the JAK2 inhibitor AG490 or the STAT3 inhibitor S3I-201 suppressed NP-induced mechanical allodynia, and the increase in IL-1β, IL-6, and TNF-α mRNA levels in CCI and SNI rats [14, 36, 59].

**Figure 1.**
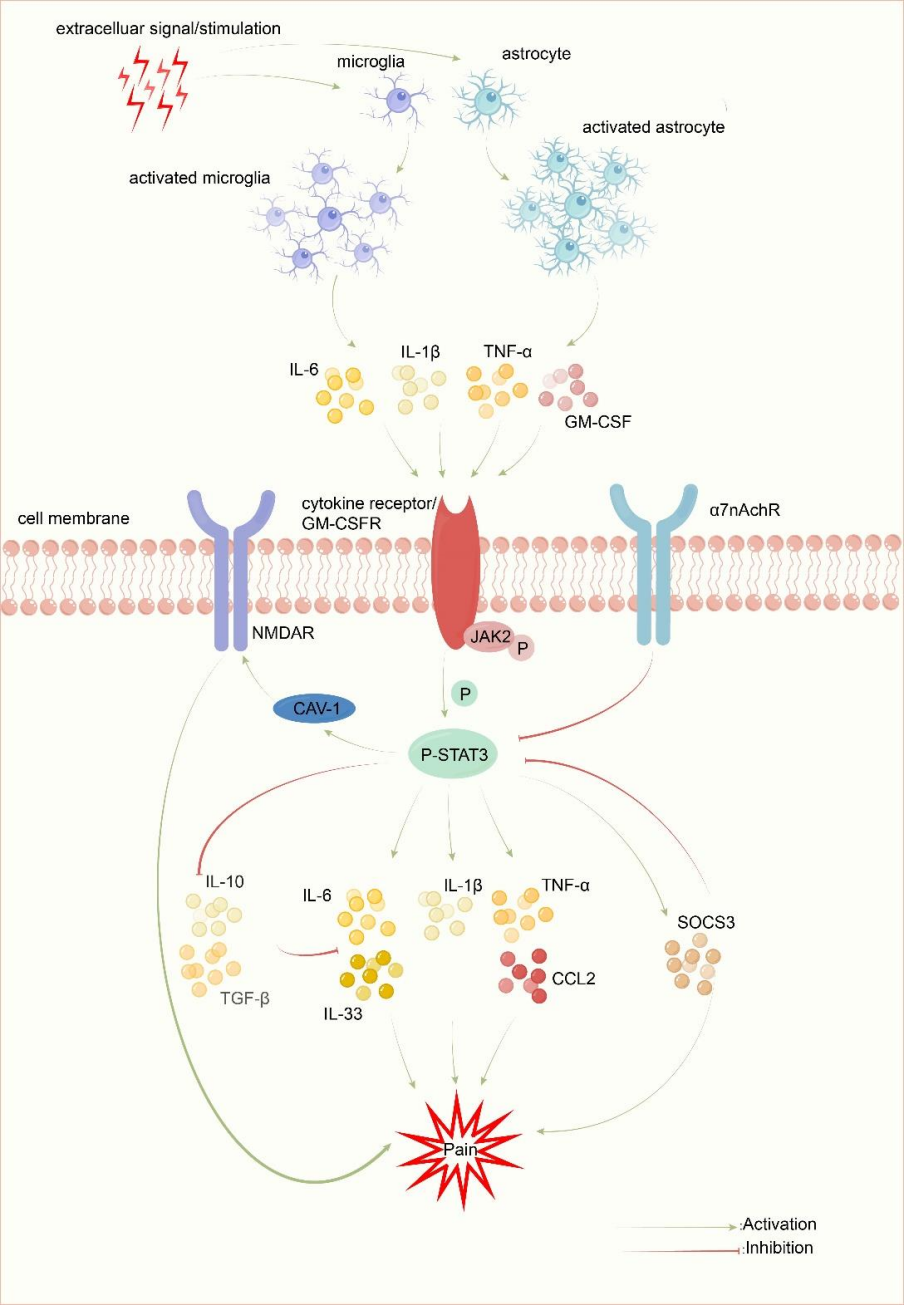
**The mechanisms of Janus Kinase 2 (JAK2)/signal transducer and activator of transcription 3 (STAT3) in chronic pain**. After extracellular signal/stimulation, the JAK2/STAT3 signaling pathway is activated by several proinflammatory cytokines and GM-CSF that are released by activated microglia and astrocytes. On the one hand, JAK2/STAT3 affects the translation and expression of IL-6, IL-1β, TNF-α, IL-33, CCL2 and SOCS3 to trigger neuroinflammatory chronic ain. On the other hand, JAK2/STAT3 promotes the NMDAR pathway to induce peripheral sensitization and chronic pain. However, IL-10, TGF-β, and α7nAchR can inhibit these mechanisms.

These findings demonstrate the promising therapeutic potential of the JAK2/STAT3 inhibitors in animals with NP. As more and more credible evidence continues to emerge, the clinical application potential of the JAK2/STAT3 therapy has also gradually garnered significant attention in the treatment of chronic pain, such as osteoarthritis (OA) and tumor pain. OA, a degenerative joint disease, presents with symptoms such as pain, stiffness, and reduced mobility owing to the loss of cartilage and bone quality at the interface of the synovial joint. Although medication [62, 63] and surgery [64] are the main treatments, their efficacy and safety remain ambiguous. Currently, treatments using regenerative methods have been established and are promising in the early treatment of cartilage degeneration in OA joints despite their failure to address inappropriate subchondral bone regeneration [64]. Research showed that OA progression could be delayed by the inhibition of subchondral TGF-β which is the downstream regulator of the JAK2/STAT3 [65]. Mima et al. confirmed that IL-6 is involved in multiple pathological processes during the initiation of OA. The JAK2/STAT3 signaling is a major pathway triggered by IL-6. They experimentally demonstrated that the JAK2/STAT3 inhibitor AG490 protects against OA progression and improves OA prognosis by reducing cartilage degeneration and arthritis pain [66].

Moreover, WP1066 is a small-molecule inhibitor that targets the JAK2/STAT3 pathway and is currently undergoing clinical trials for its application against various brain tumors in children and adults. It is effective against various cancer cells and mouse models. Allaf et al. specifically narrowed down their research to schwannomatosis-induced intractable chronic neuro-pathic pain. They identified that WP1066 reduced cell viability and STAT3 phosphorylation and induced the expression of markers for both necroptosis and caspase-dependent cell death in the schwannomatosis cell line. These findings suggest that WP1066 holds promise for providing relief from chronic pain in animal models [67]. The mechanisms of Janus Kinase 2 (JAK2)/signal transducer and activator of transcription 3 (STAT3) in chronic pain are illustrated in Figure 1.

## 7. Concluding remarks and future perspectives

In this review, we outlined the mechanism by which JAK2/STAT3 triggers chronic pain. We also review the therapeutic potential of pharmacological inhibitors of the JAK2/STAT3 for different types of chronic pain. These results suggest that the JAK2/STAT3 signaling has a critical effect on the development and maintenance of chronic pain. Nevertheless, these observations provide a basis for further research and exploration.

From the perspective of experimental design, most studies have solely used male rodents, which reduces the overall credibility of the role of the JAK2/STAT3 signaling in chronic pain. Considering the critical sex-related differences in the role of microglia in pain, future studies should include female rodents [41]. However, among these studies, the discrepancy in the effect of the JAK2/STAT3 may stem from multiple factors, such as the use of different antibodies and dosages or variation in the time windows of observation [33]. It is also possible that differences in animal models, injection sites, and behavioral tests influence the effects of drugs on chronic pain[30, 37]. However, they may share similar pathogenesis and potential mechanisms. Thus, future studies should investigate the effects of the JAK2/STAT3 using different models and experimental designs to provide more comprehensive insights.

From the perspective of mechanism research, Xue et al. surmised that the activation of the JAK2/STAT3 signaling pathway is time-dependent in bCCI rat models, which may be postponed in different cell types [11]. Wan et al. revealed that a discrepancy in the time course of gene and protein expression or post-transcriptional regulation may cause an inconsistency between the gene and protein expression levels of IL-6, JAK2, and STAT3 [7]. Further studies are required to elucidate the mechanisms underlying the JAK2/STAT3 pathway in different stages of chronic pain. Furthermore, few studies have addressed the downstream genes of the JAK2/STAT3 signaling and the effect of central sensitization and neuropathic pain in the RN, which is of great importance to explore [36-39, 59].

From perspectives of therapeutic potential, Cheppudira et al. speculated that AG490 was more effective in reducing mechanical allodynia than in reducing mechanical hyperalgesia [24]. This finding potentially indicates that the analgesic potency may differ from the pain model changes. Therefore, whether the JAK2/STAT3 inhibitors exert similar analgesic effects in different types of chronic pain requires further investigation. Blockade of the JAK2/STAT3 signaling pathway via specific inhibitors attenuates various types of chronic pain; however, the underlying mechanisms remain elusive, such as the non-opioid mechanism of AG490 [24, 30]. Therefore, additional studies are necessary to investigate the therapeutic potential of the JAK2/STAT3 in the treatment of chronic pain.

From perspectives of new therapy development and clinical application, we anticipate that the findings of the aforementioned experiments will aid the development of new treatments for early OA and could either negate or delay the need for joint replacement. Moreover, this will reduce the financial burden on healthcare providers and the patients themselves. Moreover, a similar mechanism has been observed in intervertebral disc degeneration(IDD) [68]. However, whether WP1066 can relieve tumor-induced chronic pain in animal models or clinical trials is yet to be explored. However, the effects of inhibitors on cytokines and other factors in the aforementioned experiments may interfere with normal physiological activities of the body. Further studies are required to investigate the therapeutic potential of the JAK2/STAT3 inhibitors in chronic pain.
